# Supplementary material for: ART is key to clearing oncogenic HPV genotypes (HR-HPV) in anal mucosa of HIV-positive MSM
Source: PLoS One. 2019 Oct 24;14(10):e0224183. doi: 10.1371/journal.pone.0224183 (PMC6813028; doi:10.1371/journal.pone.0224183)
Supplement: Attachment — Submitted filename: PONE-D-19-18194.docx [file pone.0224183.s002.docx]

**PONE-D-19-18194**

**General**

This is a very important study exploring the natural history of anal HPV infection and anal dysplasia among HIV+ positive MSM. The study was well executed and has robust analysis. It also explored treatment of HSILs with a variety of methods including impact of giving the quadrivalent vaccine. I recommend the manuscript for publication after the comments highlighted below are addressed.

**Methods:**

**Comment 1: Design: 5 paragraph last sentence:**

*“At the same visit, biopsies were taken using an endoscopic retrograde cholangiopancreatography catheter.”*

What biopsies were these? Anal or liver?

**Comment 2: Definition of variables**

*“Histology with anal HSIL+: from HSIL to ASCC.”*

*“Histology with LSIL+: from LSIL to ASCC.”*

These are not clear and need to be updated.

**Results**

**Comment 3: 5. HPV genotype distribution by histology**

Text in this section and reference to the tables needs to be clarified.

For example the second paragraph *“As depicted in Figure 1, none of the 36 HPV genotypes isolated in normal mucosa were significantly associated with this histological finding (normal mucosa), and the most frequently isolated genotypes were 6 (15.4%), 16 (21.6%), 42 (18.5%), 45 (23.2%), and 62 (31.9%).”*

**Comment 4:** The information on the most common genotypes in actually in Figure 2 not figure 1 which shows the stabilisation of the HSIL+ rates

**Comment 5:** Again the third paragraph, **“***In LSIL+ mucosa samples, the most frequently isolated genotypes were 6 (19.1%), 11 (19.8%), 16 (32.3%), 31 (16.7%), 42 (19.1%), and 55 (17.3%), as shown in Figure 2. Genotype 66 was significantly associated with LSIL (12.9% vs. 5.6% p=0.001) (Figure 1).”*

It is not immediately clear from Figure 1 that type 66 is associated with LSIL

**Comment 6: 6. Correlation of anal cytology and HPV PCR with histological findings**

“The cytology study showed that 165 samples were normal and 232 had some degree of

dysplasia. Among the patients with dysplastic samples, 36.6 % had normal anoscopy

(p=0.0001), 50% LSIL (p=0.0001), and 17.2% HSIL (p=0.001).” This section is difficult to follow for the readers. For example you would need to calculate that (85/232)=36.6 had normal. There is another % i.e..43.4% which is next to 85 and can be confusing to the reader.

**Comment 7: Table 1:** As with other abbreviations, can the authors put the full name for the SDU in table footer

**Comment 8: Table 5:** Needs a bit of formatting so that all the text and number are of the same **font.**

**Discussion**

**Comment 9: Second paragraph.** This sentence is missing the 95% CI *“The duration of ART was the sole factor favoring oncogenic virus clearance (OR 1.016; 95% CI),”*

**Comment 10: Third paragraph.** It is this sentence which explains what Figure 1 showed *“However, we have observed a significant reduction in the HSIL+ rate among HIV+ MSM since the start of the program in 2010, with a stabilization since 2016”*

However, this is never mentioned in the results section. It then ties with observation and comments in the results section that the part 5 and 6 of the results needs to be revised as they are rather confusing to the reader.

**Comment 11: Fourth paragraph.** The finding on nadir CD4+ is important *“Factors related to infection with HPV oncogenic virus were a history of CD4 nadir < 200 cells/uL (OR: 2.363, 95% CI: 1.062-5.256), and simultaneous infection by low- and highrisk HPV genotypes (OR: 1.842; 95% CI: 1.015-3.344).”*

Can the authors mention that this confirms previous findings and cite some few references

**Comment 12: Limitations paragraph:** The spelling for HIV needs to be updated *“A major study strength is that it reports on the first prospective cohort of VIH+ MSM, demonstrating the value of implementing a program to screen for, diagnose, treat, and follow up HSIL+ lesions.”*
