# Supplementary material for: ART is key to clearing oncogenic HPV genotypes (HR-HPV) in anal mucosa of HIV-positive MSM
Source: PLoS One. 2019 Oct 24;14(10):e0224183. doi: 10.1371/journal.pone.0224183 (PMC6813028; doi:10.1371/journal.pone.0224183)
Supplement: Attachment — Submitted filename: Responses revised1019.docx [file pone.0224183.s003.docx]

We are grateful to the reviewers for their valuable comments, which have all been taken into account in our revision, as explained in our point-by-point responses.:

Reviewer #1: General
This is a very important study exploring the natural history of anal HPV infection and anal dysplasia among HIV+ positive MSM. The study was well executed and has robust analysis. It also explored treatment of HSILs with a variety of methods including impact of giving the quadrivalent vaccine. I recommend the manuscript for publication after the comments highlighted below are addressed.

Methods:
1- Comment 1: Design: 5 paragraph last sentence:
“At the same visit, biopsies were taken using an endoscopic retrograde cholangiopancreatography catheter.”
What biopsies were these? Anal or liver?

We have now specified in the revised text that we took biopsies of the anal mucosa, as follows:

“At the same visit, biopsies of the anal mucosa were taken using an endoscopic retrograde cholangiopancreatography catheter”.

2- Comment 2: Definition of variables
“Histology with anal HSIL+: from HSIL to ASCC.”
“Histology with LSIL+: from LSIL to ASCC.”
These are not clear and need to be updated.

We have clarified these definitions.

Results
3- Comment 3: 5. HPV genotype distribution by histology
Text in this section and reference to the tables needs to be clarified.
For example the second paragraph “As depicted in Figure 1, none of the 36 HPV genotypes isolated in normal mucosa were significantly associated with this histological finding (normal mucosa), and the most frequently isolated genotypes were 6 (15.4%), 16 (21.6%), 42 (18.5%), 45 (23.2%), and 62 (31.9%).”

We have clarified the text and reference accordingly in the revised Results section.

4-Comment 4: The information on the most common genotypes is actually in Figure 2 not figure 1 which shows the stabilisation of the HSIL+ rates.

We apologize for this typographic error, which has been amended.

5- Comment 5: Again the third paragraph, “In LSIL+ mucosa samples, the most frequently isolated genotypes were 6 (19.1%), 11 (19.8%), 16 (32.3%), 31 (16.7%), 42 (19.1%), and 55 (17.3%), as shown in Figure 2. Genotype 66 was significantly associated with LSIL (12.9% vs. 5.6% p=0.001) (Figure 1).”
It is not immediately clear from Figure 1 that type 66 is associated with LSIL

We are again grateful to the reviewer for detecting these errors. These sentences now read as follows:

In LSIL+ mucosa samples, the most frequently isolated genotypes were 6 (19.1%), 11 (19.8%), 16 (32.3%), 31 (16.7%), 42 (19.1%), and 55 (17.3%), as shown in Figure 2B. Genotype 66 was significantly associated with LSIL (12.9% vs. 5.6% p=0.001).

6- Comment 6: 6. Correlation of anal cytology and HPV PCR with histological findings
“The cytology study showed that 165 samples were normal and 232 had some degree of dysplasia. Among the patients with dysplastic samples, 36.6% had normal anoscopy (p=0.0001), 50% LSIL (p=0.0001), and 17.2% HSIL (p=0.001).” This section is difficult to follow for the readers. For example you would need to calculate that (85/232)=36.6 had normal. There is another % i.e..43.4% which is next to 85 and can be confusing to the reader.

We have clarified the text accordingly, making these results easier to follow.

7- Comment 7: Table 1: As with other abbreviations, can the authors put the full name for the SDU in table footer.

This has been done.

8- Comment 8: Table 5: Needs a bit of formatting so that all the text and number are of the same font.

This has been done.

Discussion
9- Comment 9: Second paragraph. This sentence is missing the 95% CI “The duration of ART was the sole factor favoring oncogenic virus clearance (OR 1.016; 95% CI),”

This typographic error has now been amended.
10- Comment 10: Third paragraph. It is this sentence which explains what Figure 1 showed “However, we have observed a significant reduction in the HSIL+ rate among HIV+ MSM since the start of the program in 2010, with a stabilization since 2016”
However, this is never mentioned in the results section. It then ties with observation and comments in the results section that the part 5 and 6 of the results needs to be revised as they are rather confusing to the reader.

- This information is given in the Part 2 of Results under the heading “Clearance and acquisition of HR-HPV infection and prevalence of HSIL+ in anal mucosa….”: However, as depicted in Figure 1, we found significant reductions in HSIL+ cases between 2010 and 2018 (42.9% vs. 4.1%, p=0.034), between 2010 and 2013 (42.9% vs. 13.8%, p=0.003), and between 2013 and 2016 (13.8% vs. 4.8%, p=0.0001), followed by a stabilization between 2016 and 2018 (4.8% vs. 4.1%, p=0.617) (Figure 1). …

We have clarified parts 5 and 6 of Results, as requested.

11- Comment 11: Fourth paragraph. The finding on nadir CD4+ is important “Factors related to infection with HPV oncogenic virus were a history of CD4 nadir < 200 cells/uL (OR: 2.363, 95% CI: 1.062-5.256), and simultaneous infection by low- and high risk HPV genotypes (OR: 1.842; 95% CI: 1.015-3.344).”
Can the authors mention that this confirms previous findings and cite some few references

This is done in the following paragraph:

“These data confirm recent observations of the protective effects ofART and CD4 levels>500 cells/uL against infection by oncogenic HPV genotypes (26). Other studies have described the presence of genital warts (27) and a deficient immune system (28) as risk factors for developing neoplasias associated with HR-HPV infection”.

12- Comment 12: Limitations paragraph: The spelling for HIV needs to be updated “A major study strength is that it reports on the first prospective cohort of VIH+ MSM, demonstrating the value of implementing a program to screen for, diagnose, treat, and follow up HSIL+ lesions.”

This has been changed to “HIV+ MSM”

With Best regards

Carmen Hidalgo Tenorio
